# A Study on the Mechanism of Selective Removal of ZERODUR Microcrystalline Glass by Polishing Abrasives in Magnetorheological Machining

**DOI:** 10.3390/ma19132879

**Published:** 2026-07-06

**Authors:** Haozheng Wang, Xiaoqiang Peng, Hao Hu, Rui Yu, Pengxiang Wang

**Affiliations:** 1 College of Intelligence Science and Technology, National University of Defense Technology, Changsha 410022, China; whz_5253@163.com (H.W.); pengxiaoqiang@nudt.edu.cn (X.P.);; 2 National Key Laboratory of Equipment State Sensing and Smart Support, National University of Defense Technology, Changsha 410073, China

**Keywords:** magnetorheological finishing, microcrystalline glass, abrasive effect, zirconium dioxide, surface roughness, non-uniform material removal

## Abstract

ZERODUR glass-ceramic is widely used in ultra-precision optical components because of its extremely low thermal expansion and excellent dimensional stability. However, its two-phase microstructure, composed of crystalline and amorphous phases with different mechanical properties, may cause non-uniform material removal during magnetorheological polishing, thereby limiting further improvement of nanoscale surface quality. To address this issue, this study investigates the effect of oxide abrasives on the surface homogenization of ZERODUR. A single-particle abrasive–workpiece contact model based on modified Hertz contact theory and elastoplastic contact analysis was established to compare the indentation responses of CeO_2_, SiO_2_, and ZrO_2_ abrasives in the two constituent phases. Magnetorheological polishing experiments were conducted under identical process parameters, and the polished surfaces were characterized by AFM over scan areas of 2 μm × 2 μm, 5 μm × 5 μm, and 10 μm × 10 μm. The results show that all three abrasives improved the surface quality of the ring-polished substrate, with ZrO_2_ achieving the best surface homogenization performance. The lowest roughness, Ra = 0.104 nm, was obtained at a 2 μm field of view, and the ZrO_2_-polished surface showed more stable roughness evolution across different scan sizes than the CeO_2_- and SiO_2_-polished surfaces. These results indicate that the elastic modulus, hardness, and mechanical compatibility of abrasives with ZERODUR play key roles in governing contact stress, indentation behavior, and final surface quality. This work addresses the lack of mechanistic understanding of abrasive-dependent surface homogenization in the magnetorheological polishing of two-phase ZERODUR glass-ceramic. The main innovation is the integration of contact-mechanics-based abrasive–workpiece modeling with multi-scale AFM characterization to clarify how abrasive mechanical compatibility affects nanoscale surface uniformity and to guide abrasive selection for ultra-smooth optical manufacturing.

## 1. Introduction

As technologies such as extreme ultraviolet lithography, advanced astronomical observation, and high-stability precision measurement continue to advance, the surface quality requirements for high-performance optical components have become increasingly stringent. Beyond geometric accuracy, sub-nanometer surface roughness, low surface defect density, and minimal subsurface damage are now key indicators for evaluating the manufacturing quality of high-end optical components [1,2]. ZERODUR microcrystalline glass is widely used in high-precision mirrors, lithography optical systems, and high-stability measurement platforms, because of its extremely low coefficient of thermal expansion and excellent dimensional stability. However, this material is a typical two-phase composite system; the crystalline and amorphous phases differ in terms of elastic modulus, hardness, and yield behavior, which can easily lead to inconsistent interphase removal during ultra-precision machining, thereby limiting further improvements in final surface quality [1,2,3].

Magnetorheological finishing (MRF) is a typical deterministic sub-micron precision machining technique. By modulating the rheological properties of a magnetorheological fluid through an applied magnetic field, it forms a flexible polishing zone with controllable stiffness and morphology on the workpiece surface, thereby achieving high-precision, low-damage, and repeatable material removal [4,5,6,7]. Previous studies have shown that MRF possesses excellent surface adaptability and stable removal characteristics, demonstrating significant advantages in the precision machining of complex optical surfaces and hard, brittle materials [5,6]. Moreover, shear stress within the polishing zone, hydrodynamic pressure, and the near-surface mechanical properties of the workpiece are considered the primary factors influencing material removal behavior [8,9,10,11]. Although research on the removal mechanism of MRF, the modeling of influencing factors, and the regulation of rheological properties is relatively extensive, there remains a lack of systematic understanding regarding the differences in contact responses of various abrasives on the surface of two-phase microcrystalline glass and their underlying mechanisms affecting the final surface quality [5,6,8,9,10].

In magnetorheological polishing systems, non-magnetic abrasives serve as the core functional phase directly involved in material removal. Previous studies have shown that abrasive type, particle size, concentration, and surface chemistry all significantly affect removal efficiency and surface roughness [8,12,13,14,15]. For optical glass processing, CeO_2_ is commonly used due to its combination of moderate chemical reactivity and mechanical action; SiO_2_ is often employed for low-damage fine finishing; and ZrO_2_, owing to its higher hardness and greater load-bearing capacity, offers potential advantages in enhancing removal stability and improving surface integrity [13,14,15,16,17]. At the same time, studies on ZERODUR and related optical glasses indicate that different oxide abrasives do not exhibit consistent performance in terms of machining efficiency, surface quality, and defect control [3]. This suggests that the mechanical matching relationship between the abrasive and the workpiece may be a key factor determining the machining results; however, this issue requires further clarification under magnetorheological polishing conditions.

Theoretically, the local contact behavior between a single abrasive grain and the workpiece surface determines the stress distribution, indentation depth, and material removal mechanism at the microscopic scale. Classical Hertzian contact theory can be used to describe elastic contact processes, while elastoplastic contact models provide a foundation for analyzing local yielding of the workpiece and material removal [18]. For two-phase materials such as microcrystalline glass, different phase regions exhibit distinct contact responses under the same external load; therefore, variations in abrasive properties may further amplify or suppress interphase removal differences. Establishing an analytical model of the abrasive–workpiece interface based on contact mechanics helps explain, at a mechanistic level, the differences in surface topography and the evolution of roughness associated with different abrasives.

Previous studies on magnetorheological finishing (MRF) of optical glasses have mainly focused on polishing-zone mechanics, removal-rate modeling, rheological regulation, and the effects of shear stress, hydrodynamic pressure, and workpiece properties on average material removal behavior [5,6,7,8,9,10,11]. These studies have provided an important basis for understanding the deterministic removal capability of MRF. However, most of them treat the workpiece as a macroscopically homogeneous material and primarily evaluate global removal rate or overall surface roughness. For two-phase glass-ceramics such as ZERODUR, the crystalline and amorphous phases have different elastic modulus, hardness, and yielding behavior. These phase-dependent properties may lead to local differences in indentation response and removal depth during polishing, but this issue has not been sufficiently addressed in existing MRF studies.

Abrasive-related studies have shown that abrasive type, particle size, concentration, slurry stability, and chemical–mechanical interactions strongly affect removal efficiency and surface quality [8,12,13,14,15,16,17]. CeO_2_ and SiO_2_ abrasives have been widely investigated in the polishing of optical glasses because of their chemical activity and fine-finishing capability, while ZrO_2_ abrasives may provide stronger mechanical support owing to their higher hardness and load-bearing capacity. Nevertheless, most available studies compare the final polishing performance of different abrasives empirically, rather than explaining how the mechanical compatibility between the abrasive and the two constituent phases of ZERODUR influences selective material removal. In particular, it remains unclear whether an appropriate abrasive can reduce the removal mismatch between the crystalline and amorphous phases under magnetorheological polishing conditions.

Here, we investigate ZERODUR microcrystalline glass as the research subject and selects three typical oxide abrasives (CeO_2_, SiO_2_, and ZrO_2_) to conduct comparative experiments under uniform magnetorheological machining parameters. First, a single-grain contact model that accounts for the elastic deformation of the abrasive grain and the elastic–plastic response of the workpiece was established based on the modified Hertz contact theory. By incorporating the typical mechanical parameters of the crystalline and amorphous phases of microcrystalline glass, the indentation depth and interphase removal differences under the action of different abrasives were analyzed; Subsequently, surface roughness and topographical features under different abrasive systems were systematically compared using multi-scale atomic force microscopy (AFM) at 2 μm, 5 μm, and 10 μm resolutions; Finally, by integrating theoretical analysis with experimental results, the influence of abrasive mechanical properties on the surface quality of magnetorheological-polished microcrystalline glass was discussed. This paper aims to provide theoretical support for the optimization of abrasive selection in microcrystalline glass magnetorheological ultra-precision machining and to provide indirect experimental support for the preparation of higher-quality ultra-smooth surfaces.

## 2. Theoretical Analysis

Magnetorheological polishing is a precision polishing technique that utilizes the rheological properties of magnetorheological fluids—smart fluids—under the influence of a magnetic field. Its core principle lies in actively and dynamically controlling the stiffness, shape, and internal motion of abrasive particles within the polishing zone by modulating the magnetic field. This section will conduct a theoretical analysis of variable-abrasive experiments from three perspectives: magnetorheological effects and rheological models, the material removal mechanism of abrasive particles, and the multi-factor coupling at the polishing interface. It will focus specifically on the core role of abrasive particles in a dynamically changing environment and compare the amount of material removed through the coupling of abrasives with the workpiece.

### 2.1. The Microscopic Mechanism of Polishing Abrasives

In the magnetorheological polishing process, the abrasive acts directly on the surface of the workpiece; therefore, under the same dispersion system, the type, particle size, and hardness of the polishing particles have a significant impact on removal efficiency and surface roughness. The hardness and particle size of the polishing abrasives are key factors affecting the surface quality of the workpiece, and both must fall within a reasonable range: if the abrasive hardness is too low, material removal is insufficient and the mechanical removal process becomes unbalanced with the chemical reaction, leading to a decline in surface quality; if the hardness is too high, although material removal efficiency increases, the mechanical removal process far exceeds the rate of the chemical reaction, making it easy to cause surface damage. If the abrasive particle size is too small, agglomeration is likely to occur, causing uneven material removal and consequently deteriorating surface quality; if the particle size is too large, over-machining may result, forming defects such as scratches on the workpiece surface, which similarly reduces surface quality. Therefore, for optimal polishing, abrasives with moderate hardness and particle size must be selected to achieve a dynamic balance between mechanical cutting and chemical corrosion, thereby balancing material removal efficiency and surface quality [19]. In this work, 50 nm-diameter abrasive particles were employed for all polishing experiments and characterizations.

First, we analyze the contact interaction between a single abrasive particle and the workpiece surface. This analysis has certain limitations. Incorporating parameters such as gravity, abrasive particle shape, and wear would substantially increase the complexity of the analysis, making it difficult for the theoretical model to achieve predictive capability. Therefore, the model is more suitable for qualitative analysis rather than quantitative prediction. Therefore, we conducted the following analysis. Using an idealized model, we examine the machining process of the abrasive particles in the polishing slurry on the workpiece surface, making the following assumptions: the effects of gravity and buoyancy on the abrasive particles are neglected; the contact between the abrasive particles and the machined surface is plastic; each abrasive particle is treated as an ideal sphere with a smooth surface and no fragmentation or wear; and the workpiece is a homogeneous, isotropic, elastoplastic semi-infinite body.

Derivation of a Hertzian elastoplastic contact model for workpiece surface correction by a rigid abrasive particle. Based on the general theory of Hertzian contact and the boundary conditions associated with a rigid abrasive particle, the key equivalent parameters are derived [18,20,21].

The governing equations used in Equations (1)–(10) are based on classical Hertzian elastic contact theory and its standard spherical-contact formulation, while the elastoplastic correction in Equation (11) is adopted from the Chang–Etsion–Bogy (CEB) contact model and related elastoplastic spherical-contact analyses [18,20,21,22,23].

To calculate the equivalent modulus of elasticity $E^{*}$, the general Hertz equivalent modulus formula is:

(1) $$\frac{1}{E^{*}}=\frac{1-v_{m}^{2}}{E_{m}}+\frac{1-v_{w}^{2}}{E_{w}}=k_{m}+k_{w}$$

where $E_{m}$ is the elastic modulus of the abrasive grain, $E_{w}$ is the elastic modulus of the workpiece; $v_{m}$ is the Poisson’s ratio of the abrasive, $v_{w}$ is the Poisson’s ratio of the workpiece; $k_{m}$ is the flexibility of the abrasive grain, $k_{w}$ is the flexibility of the workpiece.

The lower the hardness and the smaller the size of the abrasive grains, the lower the equivalent elastic modulus of the system. This directly leads to an expansion of the contact area, a reduction in the maximum contact stress, and a decrease in the effective penetration depth of the workpiece, which is the root cause of the differences in machining results between abrasive grains of varying hardness.

To calculate the equivalent radius of curvature $R^{*}$, the general Hertz equivalent curvature formula is:

(2) $$\frac{1}{R^{*}}=\frac{1}{R_{1}}+\frac{1}{R_{2}}$$

where $R_{1}=R$ is the radius of curvature of the abrasive grain, and $R_{2}\to \infty$ is the radius of curvature of the workpiece. Substituting these values yields $R^{*}=R$.

Assuming that the normal load borne by a single abrasive grain is $R^{*}$, the contact radius is:

(3) $$a={\left(\frac{3FR}{4E^{*}}\right)}^{\frac{1}{3}}$$

The lower the hardness of the abrasive grain, the smaller $E^{*}$ is, and the larger the contact radius becomes, causing the contact pressure to be more dispersed.

The maximum compressive stress at the center of the contact zone is:

(4) $$p_{\max}=\frac{3F}{2\pi a^{2}}={\left(\frac{6FE^{*2}}{\pi^{3}R^{2}}\right)}^{\frac{1}{3}}$$

The lower the hardness of the abrasive grain, the smaller $E^{*}$ is, and the smaller $p_{\max}$ becomes. As a result, it becomes more difficult for the workpiece to undergo plastic yielding and thus more difficult to achieve effective material removal.

The total normal approach is:

(5) $$\delta_{total}=\frac{a^{2}}{R}={\left(\frac{9F^{2}}{16E^{*2}R}\right)}^{\frac{1}{3}}$$

The total normal approach $\delta_{total}$ is the sum of the deformation of the abrasive grain itself and the deformation of the workpiece, namely:

(6) $$\delta_{total}=\delta_{m}+\delta_{w}$$

Because the indentation depth into the workpiece determines the material removal capability, deformation separation must be performed.

Based on the displacement field solution of Hertzian contact, the normal displacement at the center of contact is proportional to the material compliance. Since the contact stress distribution of the abrasive grain and the workpiece is exactly the same, their deformation amounts satisfy:

(7) $$\frac{\delta_{m}}{\delta_{w}}=\frac{k_{m}}{k_{w}}=\frac{\left(1-v_{m}^{2}\right)/E_{m}}{\left(1-v_{w}^{2}\right)/E_{w}}$$

The analytical formula for deformation separation is:

(8) $$\delta_{m}=\frac{k_{m}}{k_{m}+k_{w}}\cdot \delta_{total}$$

(9) $$\delta_{w}=\frac{k_{w}}{k_{m}+k_{w}}\cdot \delta_{total}$$

It is further assumed that the abrasive grain undergoes only elastic deformation, while the workpiece undergoes elastic–plastic deformation, which is the optimal engineering machining scenario. In this case, the critical yield stress of the abrasive grain is far greater than the maximum contact compressive stress, so the abrasive grain deforms only elastically without plastic blunting, while only the workpiece undergoes elastic–plastic deformation. This is the most ideal condition for lapping, grinding, and polishing. At this point, only the yield criterion of the workpiece is considered: when the maximum pressure at the center exceeds the yield stress of the workpiece, substituting into Equation (4) gives the critical yield load:

(10) $$F_{cw}=\frac{\pi^{3}p_{cw}^{3}R^{2}}{6E^{*2}}$$

The lower the hardness of the abrasive grain, the smaller $E^{*}$ is, and the greater the load required to bring the workpiece into the plastic deformation stage.

During machining, when the normal pressure exceeds the yield stress, the CEB elastic–plastic correction model is adopted on this basis to modify the conventional Hertzian contact model.

(11) $$\frac{F}{F_{cw}}={\left(\frac{\delta_{w}}{\delta_{cw}}\right)}^{1.263}\cdot \left[1-\exp\left(-{\left(\frac{\delta_{w}}{\delta_{cw}}\right)}^{1.426}\right)\right]$$

where $\delta_{cw}$ is the critical indentation depth for the initial yielding of the workpiece, representing the threshold indentation depth required for the workpiece surface to transition from purely elastic deformation to plastic yielding. Specifically, when the abrasive grain comes into contact with the workpiece, if the total approach δ (the relative displacement between the centers of the abrasive grain and the workpiece) is smaller than $\delta_{cw}$, the contact zone remains in a purely elastic state and the workpiece does not undergo permanent plastic deformation; when δ exceeds $\delta_{cw}$, the workpiece begins to yield plastically, thereby resulting in material removal. This can be obtained by substituting into Equation (9).

### 2.2. The Effect of Differences in Abrasive Hardness on Variations in Glass Removal Rates Between the Two Phases

In this experiment, three types of polishing abrasives (CeO_2_, SiO_2_, and ZrO_2_) were selected. Table 1 shows the mechanical properties of the four abrasives [17,22,23,24]:

Schott microcrystalline glass is a typical two-phase composite material, consisting of both crystalline and amorphous phases. Due to differences in microstructure and chemical bonding between the two phases, their elastic modulus, hardness, and yield behavior vary. Typically, the crystalline phase possesses a higher elastic modulus and hardness, offering greater resistance to localized indentation and plastic deformation; the amorphous phase, in contrast, is relatively “softer” and is more prone to yielding and material removal under the same load. Consequently, during magnetorheological polishing, the two phases are not removed at exactly the same rate but exhibit distinct selective removal characteristics.

**Figure 1:**
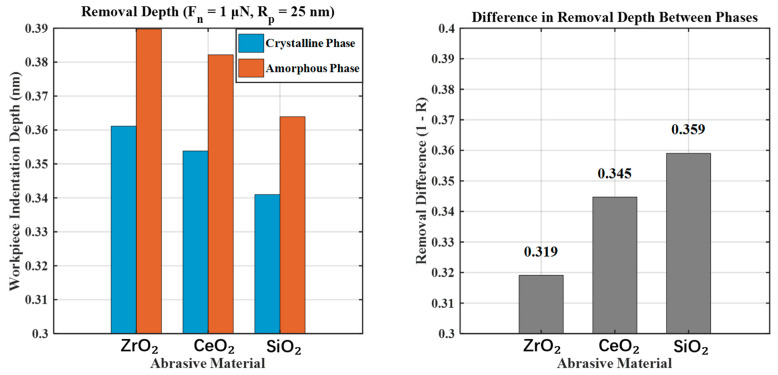
Simulated indentation depths and interphase removal differences for CeO_2_, SiO_2_, and ZrO_2_ abrasives.

Table 2 shows examples of key mechanical properties for the crystalline and amorphous phases of Schott microcrystalline glass:

To quantitatively characterize the selective removal behavior of two-phase glass under different abrasive systems, this study selected CeO_2_, SiO_2_, and ZrO_2_ for comparison. Combining typical mechanical parameters of the crystalline and amorphous phases of microcrystalline glass, numerical calculations of the indentation depth under the action of a single abrasive particle were performed using MATLAB 9.8.0.1323502 (R2020a). In the calculations, a fixed normal load of 1 μN was applied. Under identical experimental conditions, the material removal depths of the crystalline and amorphous phases acted upon by different abrasives were calculated separately. Furthermore, a ratio of removal differences between the two phases was defined to characterize the uniformity of material removal. The simulation results for removal differences are shown in Figure 1.

The numerical results indicate that there are significant differences in the removal capabilities of different abrasives for crystalline and amorphous phases. The image on the left shows the removal depths of the microcrystalline glass phase and the amorphous phase under the same fixed load; orange indicates the removal depth of the crystalline phase, and blue indicates the removal depth of the amorphous phase. The image on the right shows a comparison of the removal difference (1 − R), where R is the ratio of the removal depths of the amorphous phase to the crystalline phase; the larger the R value, the smaller the difference. Overall, as the stiffness and load-bearing capacity of the abrasive increase, the removability of the crystalline phase improves, and the difference in removal depth between the two phases gradually decreases; conversely, when the abrasive is softer or exhibits greater contact compliance, the phenomenon of preferential removal of the amorphous phase becomes more pronounced, and the unevenness in removal between the two phases intensifies. These model results suggest that, in the magnetorheological polishing of two-phase microcrystalline glass, abrasive selection not only affects the overall removal efficiency but may also influence the uniformity of phase removal at the microscopic scale.

## 3. Results and Discussion

### 3.1. Experimental Design and Evaluation Methods

The material used is ZERODUR from SCHOTT’s microcrystalline glass series. The CTE (Coefficient of Thermal Expansion) class is SCHOTT’s classification of the linear average thermal expansion coefficient of ZERODUR microcrystalline glass within the standard temperature range of 0 °C to 50 °C, reflecting the material’s dimensional stability under temperature fluctuations. The microcrystalline glass used has a CTE rating of Expansion Class 0 EXTREME (0 ± 0.007 × 10^−6^/K). This material features an extremely low coefficient of thermal expansion and excellent dimensional stability, making it widely used in high-end optical applications such as EUV lithography objectives, large-aperture astronomical mirrors, and high-stability precision measurement systems. Consequently, it imposes extremely high requirements for surface topography and sub-nanometer roughness control.

**Figure 2:**
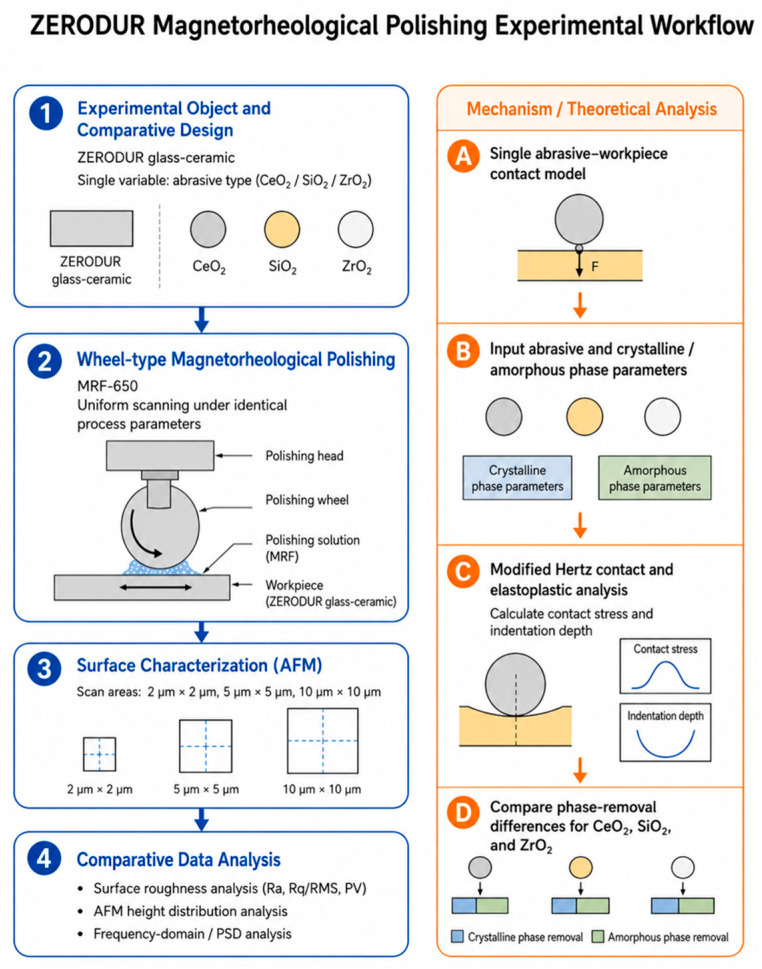
Experimental workflow.

To investigate the effect of abrasive type on the uniformity and surface quality of magnetorheological polishing, this study employed a single-factor comparative experiment. While keeping the formulation of the polishing slurry, magnetic field strength, wheel speed, flow rate, and penetration depth constant, only the type of abrasive was varied to ensure the uniqueness of the experimental variable. The experimental workflow of this study is presented as follows (Figure 2).

Commonly used abrasives include oxides (CeO_2_, Al_2_O_3_, SiO_2_), carbides (SiC, B_4_C), and superhard materials (diamond). Taking into account the material properties of the workpiece, removal efficiency, and surface quality requirements, polishing slurries were prepared using three oxide abrasives (CeO_2_, SiO_2_, and ZrO_2_) and applied to microcrystalline glass samples that had undergone ring polishing under identical process conditions for uniform polishing. In the ultraprecision magnetorheological machining process, abrasive particle size and the abrasive system are the key factors in controlling surface quality; the use of fine-grained abrasives can significantly optimize surface smoothness and flatness [3,13,15,25,26,27]. After machining, atomic force microscopy was used to measure surface topography within three field-of-view ranges (2 μm, 5 μm, and 10 μm), and parameters such as Ra, Rq (or RMS), and PV were selected for a comprehensive evaluation of the machined surface. Among these, Ra and Rq characterize the amplitude of microscopic surface undulations, while PV reflects the characteristics of local extreme undulations; together, these three parameters allow for an evaluation from different perspectives of the influence of the abrasive system on surface leveling capability and the tendency to introduce defects.

This experiment utilized the MRF-650 magnetorheological polishing machine developed by the National University of Defense Technology (Changsha, China). Building upon the university’s proprietary polishing fluid, the experiment ensured the uniqueness of experimental variables by altering only the polishing abrasive particles.

**Figure 3:**
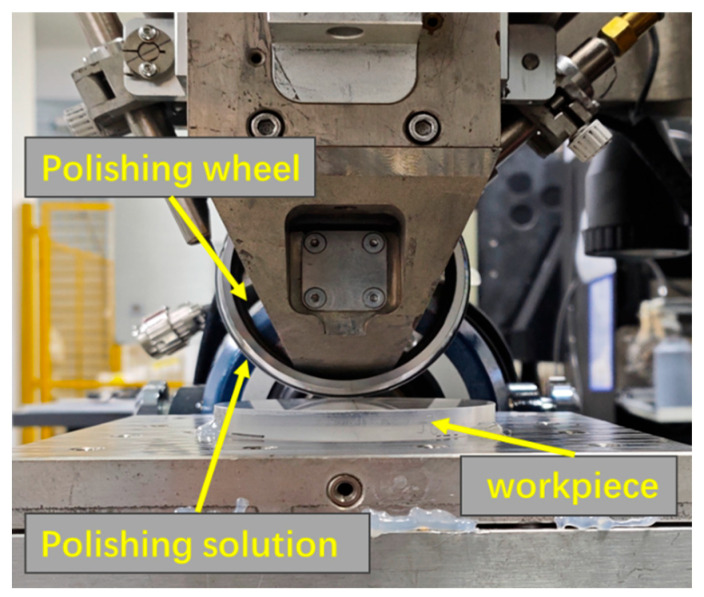
Experimental Processing.

Figure 3 shows the actual polishing process.

The reference parameters for the magnetorheological application are as follows: a 100 mm polished wheel, a rotational speed of 235 rpm, a flow rate of 100, a magnetic field current of 7A and a polishing depth of 0.15 mm. Following the experimental processing, the primary focus is on high-frequency roughness and the surface removal status as characterized by atomic force microscopy.

This study characterizes the surface topography using atomic force microscopy (AFM), focusing on height distribution, frequency-domain features, and root-mean-square (RMS) roughness. The height distribution is used to evaluate surface uniformity, while frequency-domain analysis reveals spatial periodicities and residual high-frequency features. RMS roughness provides a quantitative measure of surface height fluctuations, enabling a comprehensive assessment of the processed surface quality.

**Figure 4:**
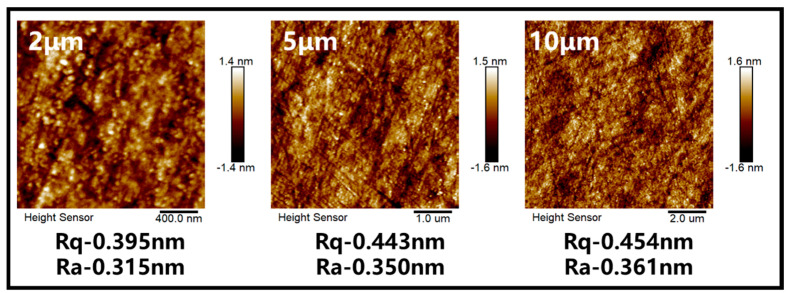
Surface quality of unprocessed workpieces.

The workpieces used in the experimental machining were Schott microcrystalline glass that had undergone a single ring polishing cycle. To determine whether magnetorheological polishing could improve surface quality, surface roughness was measured at three different ranges—10 μm, 5 μm, and 2 μm—yielding the results shown in Figure 4.

Using substrates with a surface roughness of approximately 0.4 nm RMS after ring polishing as the research subject, we first investigated the effectiveness of different polishing abrasives in improving surface roughness. Then, we used a magnetorheological polishing system to conduct comparative experiments using polishing slurries formulated with three types of abrasives (CeO_2_, SiO_2_, and ZrO_2_) and performed uniform sweeping polishing on the substrates under identical processing parameters.

The prepared polishing slurry used abrasive particles with a nominal size of 50 nm. The polishing system was a water-based slurry containing commonly used additives, including dispersants, lubricants, and chelating agents, such as glycerol and PEG. For the Bruker atomic force microscope used in this study, the short-term repeatability of Z-direction height or roughness measurements is typically on the order of the Z-noise level, several tens of picometers RMS.

### 3.2. Surface Roughness Results for Different Abrasives

In the field of precision optics, surfaces with a surface roughness (Ra) value of less than 0.3 nm are typically defined as ultra-smooth surfaces. Thanks to their extremely low roughness and undamaged, high-quality surface structure, such surfaces can significantly reduce optical scattering losses and are an indispensable fundamental performance metric for high-end core optical components such as lithography objectives and X-ray mirrors [1,2].

The experimental results indicate that different abrasive systems produce significant variations in the magnetorheological polishing performance of ZERODUR microcrystalline glass. Overall, all three abrasives were able to further reduce surface roughness compared to the original ring-polished substrate, demonstrating that magnetorheological polishing is highly effective at correcting high-frequency surface irregularities; however, the performance of the different abrasives varied in terms of removal uniformity and surface leveling capability.

After polishing, the surface roughness of the surfaces processed with the four abrasives was measured using an atomic force microscope, with the atomic force field range divided into three intervals: 10 μm, 5 μm, and 2 μm.

Processing using cerium oxide (CeO_2_):

CeO_2_ and SiO_2_ abrasives exhibit similar machining results, and both can significantly improve the surface quality of the initial substrate. Under a 2 μm scanning field of view, the surface Ra of both abrasives after polishing reached 0.2 nm, with Ra decreasing by approximately 35% compared to the initial substrate; when the field of view was expanded to 10 μm, Ra remained stable at 0.25 nm, and Ra decreased by over 30% compared to the initial substrate, achieving the preparation of high-quality sub-nanometer ultra-smooth surfaces.

**Figure 5:**
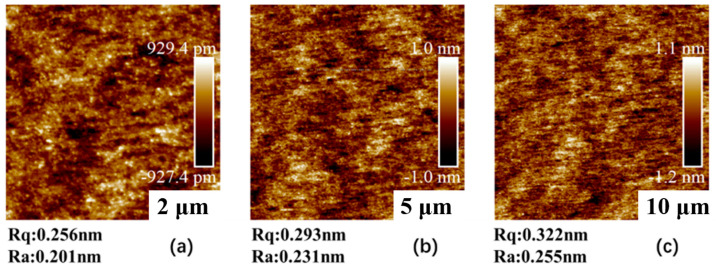
(**a**) 2 μm field of view (**b**) 5 μm field of view (**c**) 10 μm field of view.

Figure 5 shows the AFM surface topography of CeO_2_-polished workpieces measured under three different fields of view.

**Figure 6:**
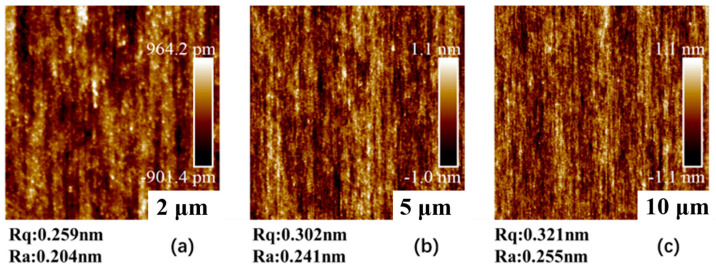
(**a**) 2 μm field of view (**b**) 5 μm field of view (**c**) 10 μm field of view.

Figure 6 shows the AFM surface topography of SiO_2_-polished workpieces measured under three different fields of view.

Processing using silicon dioxide (SiO_2_):

The surface topography of both abrasives after polishing is highly consistent. As shown in the AFM topography images, both surfaces exhibit only uniform, oriented cutting patterns resulting from the shear movement of the abrasive during the magnetorheological polishing process, with no discrete machining defects or extreme peaks and valleys. Across the entire field of view, the Rq/Ra ratio remains stable between 1.25 and 1.28. The surface height distribution follows a Gaussian random distribution, and roughness exhibits a linear, steady change as the field of view expands. The excellent scale self-similarity of the topography indicates that the material removal characteristics and surface leveling effects of the two methods are highly consistent.

**Figure 7:**
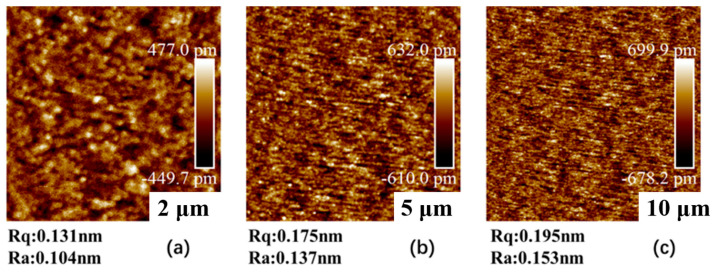
(**a**) 2 μm field of view (**b**) 5 μm field of view (**c**) 10 μm field of view.

Figure 7 shows the AFM surface topography of ZrO_2_-polished workpieces measured under three different fields of view.

Machining with zirconia (ZrO_2_):

Across the entire scanning field of view, ZrO_2_ abrasives demonstrate superior overall polishing performance compared to CeO_2_ and SiO_2_. The Ra value across the entire field of view decreased by more than 60% relative to the initial surface, while the Rq value showed a maximum reduction of 66.8% compared to the initial substrate—nearly double the reduction in surface roughness achieved with CeO_2_ and SiO_2_ processing. The measured surface roughness Ra reached 0.104 nm. Even within a 10-μm field of view, the surface maintains very low roughness values, suggesting that ZrO_2_ not only improves local micro-roughness but also exhibits stronger cross-scale surface homogenization behavior; this trend is consistent with a reduced tendency for phase-selective removal between the crystalline and amorphous phases. These results suggest that, under current magnetorheological machining conditions, ZrO_2_ abrasives may more effectively balance material removal capacity with surface integrity control, making them a promising abrasive system for optical components with extreme precision requirements, such as EUV lithography objectives.

The ZrO_2_-polished surface exhibits more uniform and denser nanoscale topographical features, with no visible scratches, pits, or isolated peaks and valleys, and the roughness parameters remain more stable across different fields of view. This suggests that the material removal process is not limited to the selective leveling of local peaks and is consistent with more continuous and uniform surface reconstruction over a larger scale.

**Figure 8:**
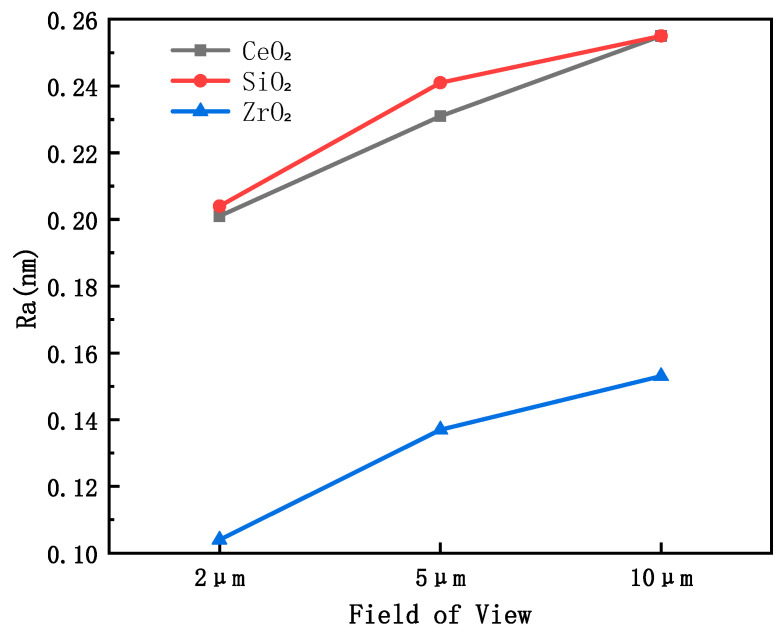
Comparison Chart of Root Mean Square (Ra) Values.

Figure 8 shows the roughness comparison curves for the three abrasives under different field-of-view conditions.

Theoretical analysis indicates that the local contact state between abrasive particles and the workpiece surface during magnetorheological polishing determines material removal behavior and its uniformity. Based on the modified Hertz contact theory and the elastoplastic contact model, it is known that different abrasives, due to variations in elastic modulus, hardness, and interfacial mechanical matching, result in differences in contact stress, indentation depth, and removal capacity between crystalline and amorphous phases, thereby exhibiting distinct interphase selective removal characteristics. Simulation results show that SiO_2_ abrasives, due to their lower load-bearing capacity, are more likely to cause preferential removal of the amorphous phase, resulting in significant differences in phase-specific removal. CeO_2_ abrasives exhibit performance intermediate between the two, offering limited improvement in removal uniformity. In contrast, ZrO_2_ abrasives are capable of reducing the difference in removal depth between crystalline and amorphous phases while ensuring effective removal, demonstrating superior phase-cooperative removal capability.

The experimental results agree well with the analytical trends described above. AFM characterization indicates that although both CeO_2_ and SiO_2_ abrasives can reduce surface roughness to the sub-nanometer level, the polished surfaces still exhibit predominantly oriented shear textures. In contrast, the ZrO_2_ abrasive exhibited lower roughness and a more stable morphological evolution trend across different field-of-view scales, achieving the lowest Ra value of 0.104 nm at a 2 μm field of view. This consistency supports the trend predicted by the contact model, namely that ZrO_2_ may reduce phase-dependent removal mismatch; however, because AFM and PSD measurements provide indirect evidence, the reduction in selective removal should be interpreted as being consistent with, rather than directly demonstrated by, the experimental results. Overall, ZrO_2_ abrasives showed the most favorable machining performance in enhancing surface uniformity and achieving an ultra-smooth surface.

### 3.3. Analysis of AFM Height Distribution and Frequency-Domain Characteristics

Selective removal features caused by differences in the removal behavior of the crystalline and amorphous phases can develop on machined glass-ceramic surfaces. Owing to the extremely small characteristic dimensions of the microstructure, the actual phase-dependent removal behavior during machining is difficult to measure directly. Therefore, AFM height distribution analysis and frequency-domain characteristics were used as indirect evidence to demonstrate the superior removal uniformity of ZrO_2_ abrasives.

This section compares the AFM-derived height distribution and frequency-domain characteristics measured over a 2 μm × 2 μm field of view.

**Figure 9:**
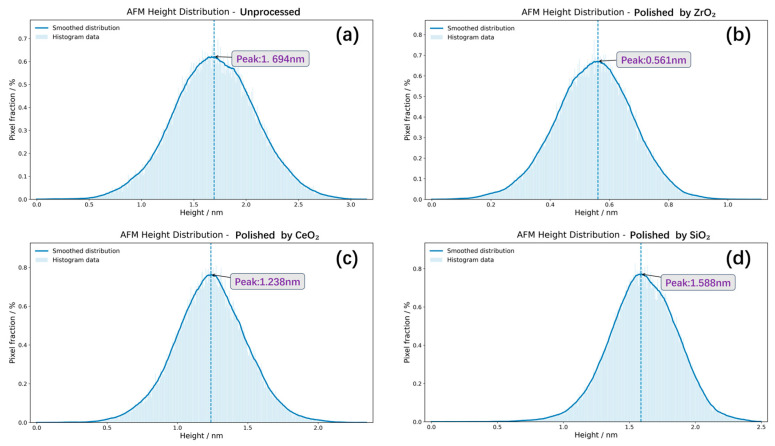
AFM height distribution profiles of different glass-ceramic surfaces: (**a**) unprocessed, (**b**) ZrO_2_-polished, (**c**) CeO_2_-polished, and (**d**) SiO_2_-polished samples.

**Figure 10:**
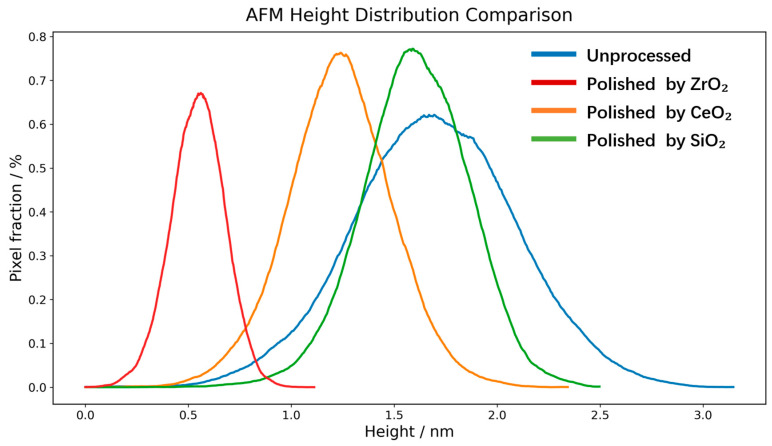
Overall comparison of AFM height distributions of glass-ceramic surfaces under different processing conditions.

Figure 9 and Figure 10 compare the AFM height distributions of different samples over a 2 μm × 2 μm field of view. The light-colored histograms represent the raw data, while the solid lines indicate the smoothed height-distribution trends. The *x*-axis denotes the surface height corresponding to the pixels in the AFM images, with a unit of nm, whereas the *y*-axis represents the percentage of pixels within a given height interval, expressed in %. As shown in the figure, the height distributions of all samples exhibit a unimodal feature, indicating that the surface heights are mainly concentrated within a specific height range.

Among the samples, the ZrO_2_-polished sample exhibits a narrower height distribution curve with a more concentrated peak, indicating that most surface pixels are confined within a smaller height range. Compared with the other samples, the ZrO_2_-polished surface shows reduced height fluctuations and a more uniform surface morphology, suggesting fewer residual height differences between peaks and valleys. This observation is consistent with reduced surface non-uniformity that may be associated with the selective removal difference between the crystalline and amorphous phases, thereby supporting improved surface quality.

**Figure 11:**
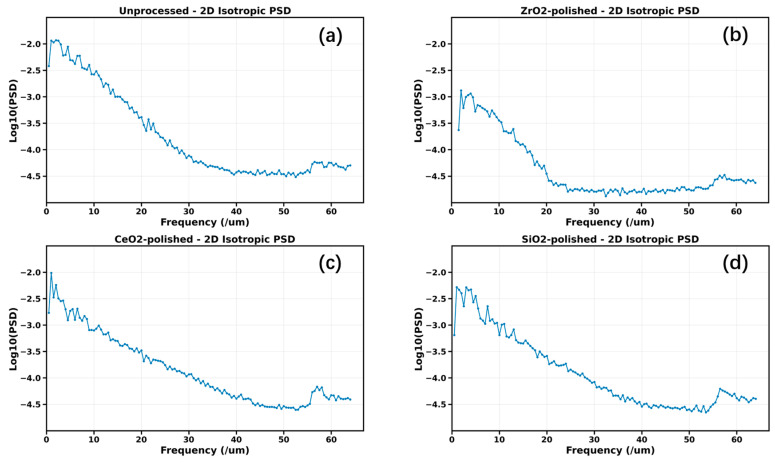
Two-dimensional isotropic PSD profiles of different glass-ceramic surfaces: (**a**) unprocessed, (**b**) ZrO_2_-polished, (**c**) CeO_2_-polished, and (**d**) SiO_2_-polished samples.

**Figure 12:**
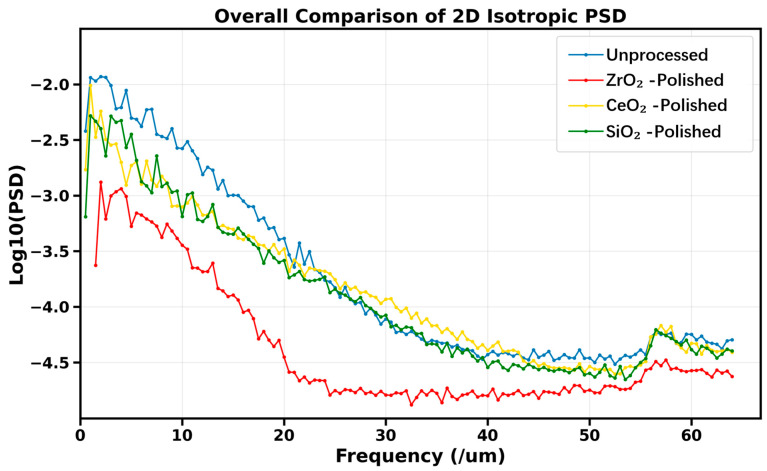
Overall comparison of the 2D isotropic PSD profiles of the unprocessed and polished glass-ceramic surfaces using ZrO_2_, CeO_2_, and SiO_2_ abrasives.

As shown in Figure 11 and Figure 12, these plots present the 2D isotropic PSD curves, which describe the intensity of surface height fluctuations at different spatial scales. In other words, they reveal the characteristic length scales that predominantly contribute to the surface roughness of each sample. The *x*-axis represents the spatial frequency, while the *y*-axis denotes the base-10 logarithm of the PSD, namely the power spectral density.

The PSD analysis results indicate that the unprocessed sample exhibits relatively high PSD values in the low- and mid-frequency regions, suggesting pronounced large-scale surface undulations and phase-scale height non-uniformity. After polishing with different abrasives, both CeO_2_ and SiO_2_ reduce the PSD intensity, with the Total Power and phase-scale Power decreasing by approximately 60%. However, relatively high power spectral density is still retained in the low- to mid-frequency regions, indicating that a certain degree of selective removal difference between the crystalline and amorphous phases remains. In contrast, the ZrO_2_-polished sample shows the lowest PSD level over the entire frequency range. In particular, within the phase-scale frequency band of 2–20 µm^−1^, which corresponds to the spatial frequency range associated with crystalline and amorphous phases, grains, phase-domain dimensions, or phase-boundary spacing, the Power is markedly reduced. The Total Power and phase-scale Power decrease by approximately 90%, demonstrating that ZrO_2_ can effectively suppress interphase steps and local height fluctuations caused by differences in the removal rates of the crystalline and amorphous phases. Therefore, ZrO_2_ exhibits superior surface planarization capability and processing uniformity.

## 4. Conclusions

To address the issue of inconsistent removal of crystalline and amorphous phases during magnetorheological polishing of ZERODUR microcrystalline glass—which can adversely affect surface uniformity and final machining quality—this study combined theoretical calculations with polishing experiments to analyze the removal behavior and surface evolution of the two phases under different abrasive conditions, thereby verifying the role of abrasive optimization in improving polishing performance. The main conclusions are as follows:

1. A local contact analysis model was established for the interaction between abrasive grains and the surface of ZERODUR microcrystalline glass under the influence of different abrasives, revealing differences between the crystalline and amorphous phases in terms of contact stress, indentation depth, and material removal response. The results indicate that the elastic modulus and hardness of the abrasive, as well as its mechanical compatibility with the workpiece material, are key factors influencing the coordination of material removal between the two phases.

2. Theoretical analysis and experimental comparisons of CeO_2_, SiO_2_, and ZrO_2_ abrasives indicated that different abrasives may affect the removal differences between the two phases and the resulting surface quality. Among them, the contact model predicts that ZrO_2_ abrasives reduce the calculated difference in removal depth between the crystalline and amorphous phases, and the AFM/PSD results are consistent with this trend toward improved synergistic removal of the two phases.

3. Polishing experiments demonstrated that all three abrasives can further reduce surface roughness on surfaces pre-machined by ring polishing; however, ZrO_2_ abrasives exhibited superior surface homogenization capabilities and better polishing results, achieving the lowest surface roughness of Ra 0.104 nm under a 2 μm field of view and producing a flatter, ultra-smooth surface.

4. This study demonstrates that optimizing the abrasive system from the perspective of interphase removal matching can effectively mitigate the mismatch in removal between the two phases during ZERODUR microcrystalline glass magnetorheological polishing, while significantly improving surface quality and processing uniformity. The findings provide a theoretical basis and experimental reference for abrasive selection and process optimization in the magnetorheological polishing of two-phase brittle materials.

## Figures and Tables

**Table 1:** Mechanical Properties.

Abrasive Material	Elastic Modulus	Poisson’s Ratio	Mohs Hardness
**CeO_2_**	220 GPa	0.25	6.5~7.0
**SiO_2_**	64 GPa	0.17	6.0~6.5
**ZrO_2_**	175 GPa	0.29	8.5~9.0

**Table 2:** Mechanical Properties.

Performance Indicator	Crystalline Phase	Amorphous Phase
**Elastic Modulus**	120 GPa	72 GPa
**Poisson’s Ratio**	0.20	0.17
**Mohs Hardness**	Grade 7~7.5	Grade 5.5~6

## Data Availability

The original contributions presented in the study are included in the article, further inquiries can be directed to the corresponding author.
